# NOD Dendritic Cells Stimulated with *Lactobacilli* Preferentially Produce IL-10 versus IL-12 and Decrease Diabetes Incidence

**DOI:** 10.1155/2011/630187

**Published:** 2011-06-12

**Authors:** Jean N. Manirarora, Sarah A. Parnell, Yoon-Hyeon Hu, Michele M. Kosiewicz, Pascale Alard

**Affiliations:** ^1^Department of Microbiology and Immunology, University of Louisville HSC, 319 Abraham Flexner Way, Louisville, KY 40202, USA; ^2^Division of Cellular and Gene Therapies, Center for Biologics Evaluation and Research, Food and Drug Administration, 1401 Rockville Pike HFM 725, Rockville, MS 20852, USA

## Abstract

Dendritic cells (DCs) from NOD mice produced high levels of IL-12 that induce IFN*γ*-producing T cells involved in diabetes development. We propose to utilize the microorganism ability to induce tolerogenic DCs to abrogate the proinflammatory process and prevent diabetes development. NOD DCs were stimulated with *Lactobacilli* (nonpathogenic bacteria targeting TLR2) or lipoteichoic acid (LTA) from *Staphylococcus aureus* (TLR2 agonist). LTA-treated DCs produced much more IL-12 than IL-10 and accelerated diabetes development when transferred into NOD mice. In contrast, stimulation of NOD DCs with *L. casei* favored the production of IL-10 over IL-12, and their transfer decreased disease incidence which anti-IL-10R antibodies restored. These data indicated that *L. casei* can induce NOD DCs to develop a more tolerogenic phenotype via production of the anti-inflammatory cytokine, IL-10. Evaluation of the relative production of IL-10 and IL-12 by DCs may be a very useful means of identifying agents that have therapeutic potential.

## 1. Introduction

Proinflammatory cytokines are increased during the active stages of type 1 diabetes and appear to be involved in disease development in NOD mice [1, 2]. DCs and macrophages from NOD mice have been shown to produce higher levels of proinflammatory cytokines, including IL-12 (p70) and TNF-*α* [3–5], as a result of NF*κ*B hyperactivity [6–9] by comparison to diabetes-resistant strains of mice, that is, C57BL/6, BALB/c, and NOR [6]. Moreover, myeloid DCs cultured from NOD bone marrow or spleen exhibit hyperactivation of NF*κ*B in response to several stimuli [8]. Taken together, these data strongly suggest that a defect in regulation of NF*κ*B activation exists in NOD DCs and macrophages and may play a key role in the modulation of APC function in NOD mice. Similarly, elevated IL-12 production as well as polymorphisms in the region of the IL-12 gene has also been associated with individuals at high risk for type 1 diabetes [10, 11]. The increase in IL-12 in NOD mice is particularly significant since it leads to activation of IFN*γ*-producing T cells which can mediate disease development [1, 2, 12]. Furthermore, suppression of IL-12 in NOD mice has been shown to reduce cellular infiltration in islets and prevent the development of diabetes [13]. Therefore, switching the cytokine profile of NOD DCs from proinflammatory to anti-inflammatory may be an effective strategy to abrogate activation of pathogenic Th1 cells and prevent diabetes development, since DCs have been shown to orchestrate the delicate balance between T-cell immunity and regulation in NOD mice [14].

Bacteria isolated from the normal gut flora are nonpathogenic and include the Lactobacillus species. Various strains of* Lactobacilli*, including *L. casei*, *L. reuteri,* and *L. plantarum*, have been shown to prevent diabetes [15, 16], collagen-induced arthritis [17], and colitis [18, 19] in animal models. In addition, *Lactobacilli* have been used to manage allergic diseases and are associated with improvement of various gastrointestinal diseases in humans [20, 21]. Recent studies have shown that various types of microorganisms, including bacteria, fungi, and parasites, can evade the immune system by inducing tolerogenic APC [22–24] and/or regulatory T cells [23, 25–28]. Some of these microorganisms, including *Lactobacilli*, elicit an anti-inflammatory response, at least in part, via Toll-like Receptor 2 (TLR2) ligation [26, 29, 30]. Components of microbial cell walls that target TLR2 have also been found to induce DCs from nonautoimmune-prone mice to produce anti-inflammatory cytokines and induce regulatory cells [29, 31–33]. There is a possibility, therefore, that stimulation of NOD DCs with *Lactobacilli* or TLR2 ligands could polarize them toward an anti-inflammatory phenotype that could protect against disease development. We propose that cytokine profiles, particularly IL-12 and IL-10, could be useful predictors of the ability of stimuli to induce DCs that may be used as a treatment for the prevention of diabetes. In the current study, we tested a TLR2 agonist, lipoteichoic acid (LTA), derived from *S. aureus,* and three different strains of *Lactobacilli* for their ability to switch the NOD DCs phenotype to an anti-inflammatory tolerogenic phenotype. We report in the present paper that transfer of BM-DCs induced to produce IL-10 into NOD mice delayed onset and decreased incidence of diabetes, whereas transfer of BM-DCs induced to produce IL-12 has the opposite effect, that is, an acceleration in diabetes onset and increase in incidence of diabetes.

## 2. Materials and Methods

### 2.1. Mice

Female C57BL/6 and NOD mice were obtained from Jackson Laboratory (Bar Harbor, ME) and maintained at the University of Louisville facility according to institutional animal care and use committee (IACUC) guidelines. Mice were anaesthetized with bromoethanol before euthanasia in order to harvest organs.

### 2.2. Antibodies and Flow Cytometry

FITC-anti-B7-1, FITC-anti-B7-2, PEcy7-anti-CD11b, and PE-anti-CD11c antibodies were purchased (BD Pharmingen, San Diego, CA). Cells were incubated with Fc block then labeled with antibodies for 20 min in DPBS 1% FCS, 0.1% NaNO_3_ and washed twice. Cells were analyzed by FACS using a FACScalibur (Becton Dickinson, Palo Alto, CA).

### 2.3. Generation of Bone-Marrow-Derived Dendritic Cells

Bone-marrow-derived dendritic cells (BM-DCs) were generated by culturing bone marrow cells for 12 days with Granulocyte-Monocyte Colony Stimulating Factor (GM-CSF) (PeproTech, Rocky Hill, NJ) in super complete medium containing RPMI (Mediatech, Herndon, VA) supplemented with 1% Hepes buffer (MP Biomedicals, LLC, Solon, OH), 1% sodium pyruvate, 1% L-glutamine, 1% penicillin-streptomycin, 1% nonessential amino acids (Mediatech, Herndon, VA), 0.1%  *β*2-mercaptoethanol (Sigma-Aldrich, Saint-Louis, MO), and 10% fetal calf serum (FCS) (Hyclone, Logan, UT). Briefly, 5 × 10^6^ bone marrow cells were cultured in large petri dishes (Nunc, Roskilde, Denmark) in 10 mL of culture medium containing 5 ng/mL of GM-CSF. New culture medium (10 mL) was added to the petri dishes at day 4. At days 6 and 9, half of the old cell culture medium (10 mL) was removed from each petri dish and replaced by new culture medium containing GM-CSF (5 ng/mL), and at day 12, cells were harvested.

### 2.4. *Lactobacilli* Cultures

The following *Lactobacilli* strains were used: human *Lactobacillus reuteri* DSM 2016 (DSMZ, Braunschweig, Germany), human *Lactobacillus plantarum* LP299v (PROBI, Lund, Sweden), and human *Lactobacillus casei* B255 (NIZO, Ede, The Netherlands). *Lactobacilli *were grown on deMan-Rogosa-Sharp (MRS) media (Difco Laboratories, Detroit, MI) under anaerobic conditions, and a single colony was expanded in MRS broth (Difco Laboratories, Detroit, MI) under anaerobic conditions and frozen in glycerol. *Lactobacilli *were thawed twice a week and grown for 20 hrs in MRS broth under anaerobic conditions, then expanded in large quantities until they reached the postexponential phase, that is, 12–16 hrs, depending on the strain. 

### 2.5. Dendritic Cell Stimulation and Transfer

BM-DCs were stimulated overnight with different stimuli including 1, 10, or 100 *μ*g/mL of LPS-free Lipoteichoic Acid (LTA) from *Staphylococcus aureus* (Invivogen, San Diego, CA), 100 ng/mL of LPS (Invivogen, San Diego, CA), or 10 × 10^6^ CFU/mL, 1 × 10^6^ CFU/mL (low dose) or 20 × 10^6^ CFU/mL (high dose)* Lactobacilli*. The supernatants were harvested and stored at −20°C until assayed using quantitative enzyme-linked immunosorbent assay (ELISA) kits (eBioscience, San Diego, CA) according to the manufacturer's instructions. For the *in vivo* experiments, gentamycin 10 *μ*g/mL was included in the culture containing *Lactobacilli* to eliminate any living bacteria, and the BM-DCs harvested after 24 hrs, washed twice with HBSS. 1 × 10^6^ treated BM-DCs were transferred into 6–8-week-old NOD mice (*n* = 4–11 mice/group). In some experiments, 0.5 mg/mouse of anti-IL-10R antibodies or isotype control (BioXCell, West Lebanon, NH) was injected once a week for a period of four weeks. Sera were collected 28 hrs following injection and stored at −20°C until assayed using ELISA kits (eBioscience, San Diego, CA) according to the manufacturer's instructions. Blood glucose was monitored weekly until 30 weeks of age using blood glucose strips and a blood glucose meter (Home Diagnostics, Inc., Ft Lauderdale, FL). Mice were considered diabetic when glucose levels were >300 mg/dl for two consecutive weeks.

### 2.6. Statistical Analysis

Data were analyzed using either the Student's *t*-test, Wilcoxon test, or nonparametric log-rank test. Each experiment was repeated with reproducible results 2–4 times. 

## 3. Results

### 3.1. Effect of a TLR-2 Ligand on NOD BM-DCs Activation and Phenotype

**Figure 1:**
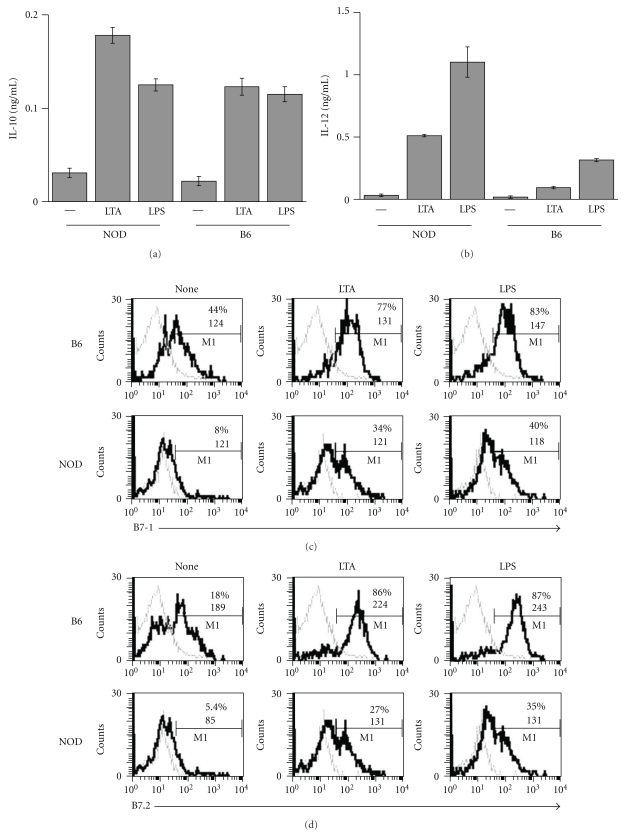
Effect of LTA on NOD and B6 DCs cytokine production and surface marker expression. BM-DCs from NOD mice were stimulated *in vitro* with 10 *μ*g/mL of LTA from *S. aureus* (LTA), 100 ng/mL of LPS, or medium alone (—or none). After 24 hrs, supernatants were collected and measured by ELISA for the presence of IL-10 (a) or IL-12 (b), or cells were collected and labeled with anti-CD11c, anti-CD11b, and anti-B7-1 (c) or anti-B7-2 (d) antibodies, and analyzed by FACS after gating on CD11b^+^CD11c^int^ cells.

Antigen presenting cells (APCs) from adult NOD mice have been shown to produce high levels of the proinflammatory cytokine, IL-12 [3–5] in response to LPS and express lower levels of costimulatory molecules such as B7-1 and B7-2 [34, 35]. The overproduction of IL-12 by NOD APC is thought to contribute significantly to disease development, since it leads to activation of IFN*γ*-producing T cells which mediate diabetes [1, 2, 12]. Because TLR2 agonists have been shown to induce production of anti-inflammatory cytokines in nonautoimmune mice and healthy human [29, 31–33], we examined whether a TLR2 ligand, lipoteichoic acid (LTA) can induce an anti-inflammatory (tolerogenic) phenotype in NOD DCs. We compared cytokine production and costimulatory molecule expression by bone-marrow-derived dendritic cells (BM-DCs) from NOD and B6 mice after stimulation with LTA isolated from *Staphylococcus aureus (S. aureus)*. DCs were cultured either in media (none), or in the presence of 10 *μ*g/mL of LPS-free LTA or 100 ng/mL of LPS. After 24 hrs, supernatants were collected and measured for the presence of the anti-inflammatory cytokine, IL-10, or the proinflammatory cytokine, IL-12, by ELISA. As reported previously, upon stimulation with LPS, NOD DCs produced considerably more IL-12 than B6 DCs (Figure 1(b)) and about the same amount of IL-10 (Figure 1(a)). Surprisingly, stimulation with LTA from *S. aureus* also induced NOD DCs to produce more IL-12 compared to B6 DCs (0.5 ng/mL versus 0.1 ng/mL) as shown in Figures 1(a) and 1(b). Although IL-10 production upon LTA stimulation was about the same or even a bit higher in NOD DCs compared to B6 DCs (0.18 ng/mL versus 0.12 ng/mL), the production of IL-12 relative to IL-10 was much greater in NOD DCs compared to B6 DCs (Figures 1(a) and 1(b)). Moreover, fewer NOD DCs expressed B7-1 and B7-2 in response to LTA and LPS compared to B6 DCs, and the level of expression of B7-2 was also lower in NOD DCs. In summary, the TLR2 ligand, LTA, had a differential effect on the cytokine profile produced by NOD and B6 DCs: LTA induced more IL-12 than IL-10 in NOD DCs while inducing the same quantity of IL-12 and IL-10 in B6 DCs.

### 3.2. Effect of LTA-Treated BM-DCs on Diabetes Development in NOD Mice

**Figure 2:**
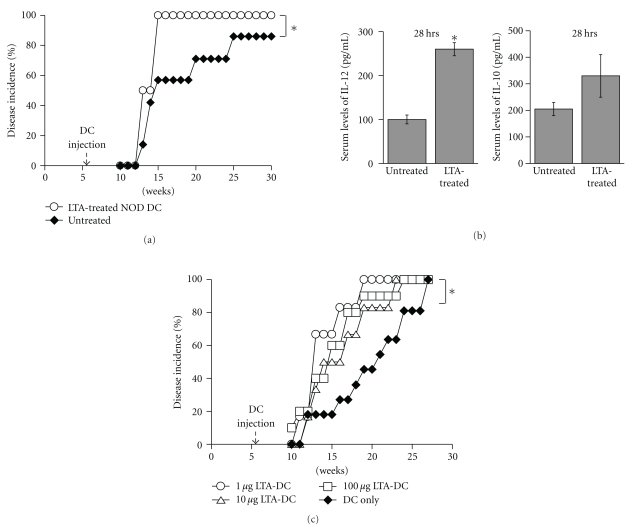
Incidence of diabetes in NOD mice injected with LTA-treated DCs. BM-DCs from NOD mice were stimulated *in vitro* overnight with 10 *μ*g/mL of LTA from *S. aureus* (LTA) or medium alone (none), washed, and one million DCs were injected into six-week-old NOD mice (*n* = 5-6), and blood glucose was monitored weekly (a). Sera from pooled NOD mice untreated or injected with DCs treated with LTA were collected at 28 hrs after injection and tested for the presence of IL-12 and IL-10 by ELISA (b). One million DCs untreated or treated with 1, 10 or 100 *μ*g/mL of LTA were injected into six-week-old NOD mice (*n* = 6–11), and blood glucose was monitored weekly (c). Mice were considered diabetic when their blood glucose levels were >300 dL/mL for 2 consecutive weeks. *indicates a significant difference from the “DC only” or “untreated” group at *P* < .01.

Since the data described above suggested that LTA from *S. aureus* promotes a more proinflammatory than tolerogenic phenotype in NOD BM-DCs, we examined the relationship between cytokine production by DCs and disease development. BM-DCs from NOD mice were cultured in the absence (none or no treatment) or in the presence of LTA from *S. aureus* (LTA-treated NOD DCs) for 24 hrs. Supernatants and cells were harvested, and supernatants tested for the presence of IL-12 and IL-10. DCs from these cultures were injected into 6-week-old NOD mice and disease incidence was assessed. Similar to the results displayed in Figure 1(a), LTA from *S. aureus* induced much more IL-12 than IL-10 production (IL-12 : IL-10 ratio of 6.6) by NOD BM-DCs (data not shown). Interestingly, 100% of NOD mice injected with LTA-treated NOD DCs developed diabetes by 15 weeks of age (Figure 2(a), open circles) indicating that a single injection of LTA-treated NOD DCs could significantly accelerate disease onset. Moreover, we found that the level of IL-12 was higher in the serum of mice injected with LTA-treated NOD DCs (Figure 2(b), left panel), whereas the level of IL-10 was not significantly different (Figure 2(b)). Because various concentrations of LTA could affect DCs differentially, we tested a lower as well as a higher dose of LTA. As shown in Figure 2(c), DCs treated with 1 *μ*g/mL, 10 *μ*g/mL or 100 *μ*g/mL of LTA and injected into young NOD mice accelerated diabetes development in a similar manner. Altogether, these data suggest that injection of LTA-treated NOD DCs may contribute to diabetes pathogenesis by producing high levels of the proinflammatory cytokine, IL-12.

### 3.3. Treatment with Different Strains of *Lactobacilli* Induces NOD DCs to Produce Different Cytokine Profiles

**Figure 3:**
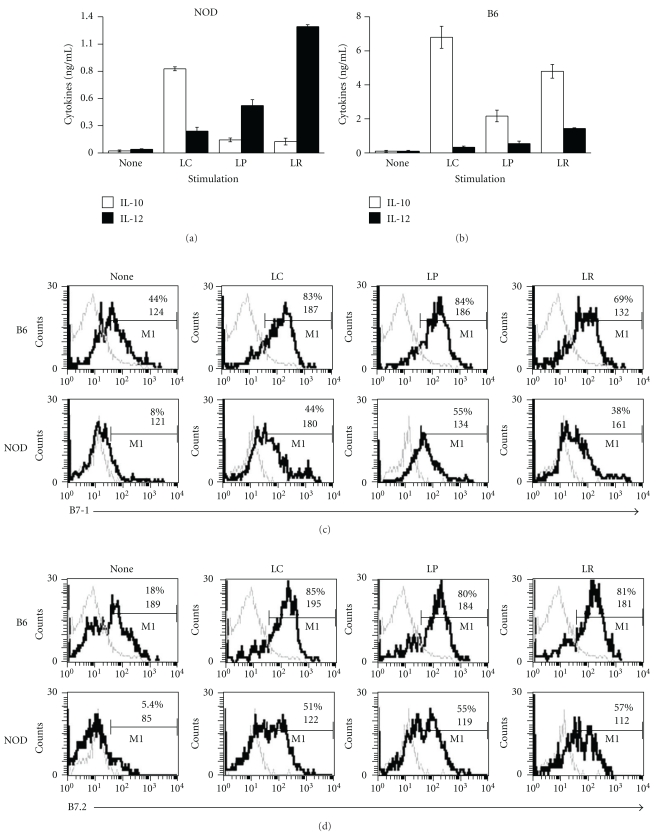
Activation of NOD and B6 DCs with different strains of *Lactobacilli in vitro*. BM-DCs from NOD or control B6 mice were stimulated for 24 hrs with 10 × 10^6^ CFU/mL *L. casei *(LC), *L. plantarum *(LP), *L. reuteri* (LR), or medium alone (none). After 24 hrs, supernatants were collected and measured by ELISA for the presence of IL-10 or IL-12 (a and b), or cells were collected and labeled with anti-CD11c, anti-CD11b, and anti-B7-1 (c) or anti-B7-2 (d) antibodies, and analyzed by FACS after gating on CD11b^+^CD11c^int^ cells.

Dendritic cells play a pivotal role in the differentiation of Th1, Th2, Th3, and regulatory T cells throughout the gastrointestinal tract [36]. *Lactobacilli* are bacteria that compose the normal flora, and feeding *Lactobacilli* has been shown to be protective in various models of autoimmune diseases, including type 1 diabetes [15, 16], collagen-induced arthritis [17], and colitis [18, 19]. It is thought that *Lactobacilli* mediate their protective effect by modulating DCs cytokine production and surface molecule expression [37]. We tested the effects of stimulating NOD and B6 DCs with three different strains of *Lactobacilli *on cytokine production and costimulatory molecule expression in order to assess whether there were any differences in the ability of these strains to induce anti-inflammatory phenotypes in NOD DCs. These three strains included* Lactobacillus casei *(*L. casei*), *Lactobacillus plantarum* (*L. plantarum*), and *Lactobacillus reuteri* (*L. reuteri*) and were selected based on their ability to induce tolerance [38] or prevent disease in animal models, including diabetes [16], arthritis [17, 39], and colitis [18, 19]. We examined the production of the proinflammatory cytokine, IL-12, and anti-inflammatory cytokine, IL-10, by NOD and B6 BM-DCs stimulated with each of the strains of *Lactobacilli* described above for 24 hrs. Interestingly, cytokine production varied dramatically depending on the strain of *Lactobacilli* used and the source of DCs, that is, NOD versus B6 mice (Figures 3(a) and 3(b)). *L. reuteri* induced NOD BM-DCs to produce the highest level of IL-12 (Figure 3(a), fourth black column) and among the lowest levels of IL-10 (Figure 3(a), fourth white column), with IL-12 produced about an 11-fold higher level than IL-10. *L. plantarum* induced a low to intermediate level of IL-12 and a very low level of IL-10 in NOD BM-DCs, and IL-12 production (Figure 3(a), third black column) was about 3-fold higher than IL-10 production (Figure 3(a), third white column). In contrast, *L. casei *not only induced NOD BM-DCs to produce the highest level of IL-10 (Figure 3(a), second white column), but also the lowest level of IL-12 (Figure 3(a), second black column), and four times more IL-10 than IL-12 was produced. Interestingly, IL-10 production was much higher, in general, in B6 DCs stimulated with all three strains of *Lactobacilli*, with *L. casei* inducing the highest levels of IL-10 and the lowest levels of IL-12 (Figure 3(b)). As shown in Figures 3(c) and 3(d), all three strains of *Lactobacilli *induced approximately the same percentages of NOD BM-DCs to express B7-1 and B7-2, but to a lower extent than B6 BM-DCs. These data suggest that the ability of *Lactobacilli* to induce an anti-inflammatory versus proinflammatory phenotype in NOD DCs varies according to the strain, and *L. casei* appears to be the only strain of the three tested that induces an anti-inflammatory phenotype. Furthermore, NOD DCs appeared to be much less predisposed to produce IL-10 in response to *Lactobacilli* compared to B6 DCs. 

### 3.4. NOD DCs Treated with Different Doses of *L. casei* Produce Different Cytokine Profiles and Induce a Different Disease Outcome When Injected into NOD Mice

**Figure 4:**
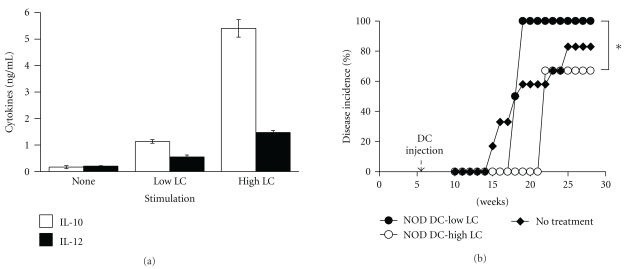
Effect of different doses of *L. casei* on the profiles of IL-10 and IL-12 production by NOD DCs *in vitro* and disease incidence. BM-DCs from NOD mice were stimulated with either 1 × 10^6^ CFU/mL (Low LC), 20 × 10^6^ CFU/mL (High LC) of the *L. casei* or medium alone (none). After 24 hrs, supernatants were collected and measured by ELISA for the presence of IL-10 (white bar) or IL-12 (black bar) (a). Six-week-old NOD mice were left untreated or received a single i.v. injection of 1 × 10^6^  DCs treated with either low (1 × 10^6^ CFU/mL-low LC) or high (20 × 10^6^ CFU/mL-high LC) dose of *L. casei* (LC) (*n* = 4–6) (b). Blood glucose was measured weekly and diabetes incidence determined. *indicates a significant difference at *P* < .05.

The dose of *Lactobacilli* has been shown to strongly influence the type of cytokine produced by DCs from B6 mice *in vitro* [37]. To determine the effect of dose of *L. casei* on cytokine production by NOD DCs, BM-DCs from NOD mice were cultured either in media (none) or in the presence of 1 × 10^6^ CFU/mL (Low-dose) or 20 × 10^6^ CFU/mL (High dose) of *L. casei*. After 24 hrs, supernatants were collected and measured for the presence of IL-10 or IL-12 by ELISA. The high dose of *L. casei* (LC) induced much more IL-10 (Figure 4(a), third white column) than the low dose of LC (Figure 4(a), second white column), and four times more IL-10 than IL-12 (Figure 4(a), third black column). These data indicate that high-dose LC is optimal for inducing NOD DCs to produce a more anti-inflammatory and tolerogenic phenotype.

We next examined whether injection of NOD DCs treated with either a high or low dose of LC into NOD mice translates into different disease outcomes. BM-DCs from NOD mice were cultured in the presence of 1 × 10^6^ CFU/mL (low LC) or 20 × 10^6^ CFU/mL of *L. casei* (high LC). Gentamycin was included in the culture medium, and no live bacteria were detected at the end of the culture (i.e., after 24 hrs). After 24 hrs, cells were harvested and injected into 6-week-old NOD mice and disease onset was monitored. In mice injected with NOD BM-DCs treated with low-dose LC, 100% of the mice develop diabetes by 18 weeks of age (Figure 4(b), closed circle). In contrast, NOD mice injected with NOD DCs treated with high-dose LC exhibited a delay in disease onset by about 4 weeks (from 17 to 21 weeks of age), as well as a significant decrease in diabetes incidence from 100% to 60% by 30 weeks (Figure 4(b), open circle). These data suggest that the dose of *Lactobacilli* used to stimulate DCs determines the cytokine profile and, consequently, disease outcome. 

### 3.5. Injection of IL-10-Producing NOD DCs into NOD Mice Decreases Diabetes Incidence

**Figure 5:**
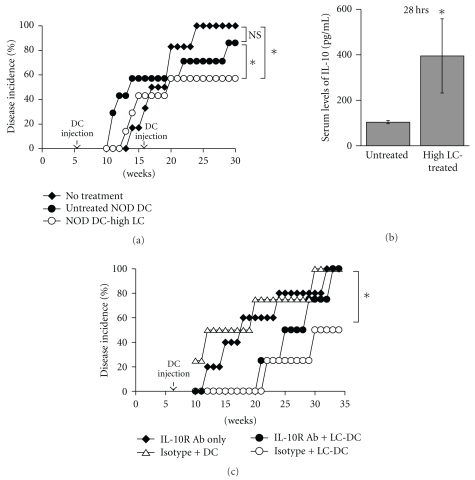
Injection of *Lactobacilli*-treated DCs into NOD mice confers some protection. Six-week-old NOD mice received two injections of 1 × 10^6^  DCs treated with high dose (20 × 10^6^ CFU/mL; NOD-high LC) of LC at 6 and 16 weeks of age (*n* = 4–6) and blood glucose was measured weekly and diabetes incidence determined (a). Sera from NOD mice uninjected or injected with DCs treated with high-dose LC (high LC) were collected at 28 hrs after injection, and tested for the presence of IL-10 by ELISA (b). Six-week-old NOD mice left uninjected or injected with 1 × 10^6^ untreated DCs (*n* = 4-5) or DCs treated with high dose of LC (*n* = 4) received anti-IL-10R or isotype control antibodies, and blood glucose was measured weekly and diabetes incidence determined (c). *indicates a significant difference at *P* < .03.

We next tested whether injections of LC-treated NOD DCs in NOD mice could actually prevent diabetes development in an IL-10 dependent manner. We administered one or two injections of NOD BM-DCs treated with high-dose LC starting at 6 weeks of age. As shown in Figures 5(a) and 5(c), injections of LC-treated DCs significantly decreased diabetes incidence by about 40% by 30 weeks in comparison to untreated NOD mice. No statistical differences were found between the untreated and untreated- DCs-injected groups, and one injection was enough to decrease disease incidence. We next examined the serum levels of IL-10 in NOD mice 28 hrs after injection of BM-DCs treated with high doses of *L. casei*. As shown in Figure 5(b), NOD mice injected with LC-treated DCs produced higher levels of IL-10 in serum 28 hrs following injection compared to noninjected NOD mice, suggesting LC-treated BM-DCs continue to produce IL-10 after injection and IL-10 may modulate the immune response *in vivo*. At 6 hrs after injection, very low levels of cytokines were detected with no differences among the different groups (data not shown), suggesting that cytokine production/accumulation requires additional time in order to allow differences to be detected. We finally tested whether the production of IL-10 by LC-treated DCs was responsible for the decrease in diabetes incidence. NOD mice were injected with anti-IL-10R or isotype control antibodies for a four-week period and LC-treated DCs. The treatment with anti-IL-10R antibodies completely abrogated production of IL-10 detected in the serum of mice injected with LC-treated DCs (data not shown). The incidence of disease was not altered in NOD mice treated with anti-IL-10R antibody only (Figure 5(c), closed diamond) compared to NOD mice injected with untreated DCs and treated with isotype control (Figure 5(c), open triangle) or anti-IL-10R antibody (data not shown). More importantly, mice injected with LC-treated DCs and treated with anti-IL-10R antibodies (Figure 5(c), closed circle) exhibited a higher diabetes incidence than mice that were injected with LC-treated DCs and treated with isotype control (Figure 5(c), open circle), indicating that IL-10 production plays an important role in LC-treated DCs-mediated protection. Altogether, these data suggest that injections of LC-treated DCs early in the disease process may be effective in preventing full-blown disease in a significant proportion of NOD mice, and IL-10 production by DCs may be useful to predict the effectiveness of the treatment.

## 4. Discussion

Previous reports have shown that feeding *Lactobacilli* has a protective effect in NOD mice [15, 16]. In the current study, we examined whether stimulation of NOD DCs with *Lactobacilli* and subsequent transfer into young NOD mice could have a similar protective effect, and whether cytokine profiles produced by DCs after stimulation could be a useful predictor of treatment efficacy. We found that stimulation of NOD DCs with three different strains of *Lactobacilli* induced upregulation of costimulatory molecules, but had differential effects on cytokine production. *L. casei* induced production of higher levels of IL-10 while *L. reuteri* and *L. plantarum* induced higher levels of IL-12, corroborating previous findings showing that different strains of *Lactobacilli* differentially modulate expression of cytokines in DCs from B6 (diabetes-resistant) mice [37]. Injection of IL-10-producing NOD DCs stimulated with *L. casei* significantly decreased disease incidence. Surprisingly, NOD DCs treated with the TLR2 agonist, lipoteichoic acid (LTA) produced large amounts of IL-12 and accelerated diabetes onset and increased disease incidence upon transfer into NOD mice. These data indicate that NOD DCs can be manipulated to become tolerogenic, and that the cytokine profile elicited can predict the ability of these cells to prevent diabetes upon injection into NOD mice.

NOD DCs have been shown to express lower levels of costimulatory molecules, such as B7 [35], and in the current study, we found that stimulation with *Lactobacilli* restored normal levels of B7-1 and B7-2 expression on the surface of NOD DCs, suggesting that *Lactobacilli* can induce NOD DCs maturation. The upregulation of B7 on NOD DCs following *Lactobacilli* treatment is particularly significant, since B7 has been found to be important for regulation of diabetes [40], and regulatory cell development and homeostasis [41, 42]. On one hand, treatment of NOD mice with anti-B7-1 antibodies accelerated diabetes development, suggesting that B7-1 may be associated with induction of regulatory cells that control diabetes development [40]. On the other hand, treatment with anti-B7-2 antibodies prevented diabetes, implicating B7-2 in disease pathogenesis [40]. There is a possibility that expression of B7-1 and B7-2 by different antigen-presenting cells play different and opposing roles. Expression of B7-1 on DCs may play a unique role in regulatory cell induction, since DCs are very potent activators of regulatory cells that can control diabetes [43, 44]. In contrast, expression of B7-2 on B cells may be very important for diabetes induction, since B cells have been shown to be crucial for diabetes development and are excellent inducers of IFN*γ*-producing T cells that respond to pancreatic antigens [45–47]. Expression of B7-2 on DCs may, therefore, not necessarily be associated with pathogenicity in NOD mice. 

Furthermore, the cytokines produced by DCs may be more crucial in determining the status of the DCs in terms of their immunogenicity versus tolerogenicity despite the expression of costimulatory molecules. We have found that a single injection of LC-treated DCs is sufficient to affect diabetes development. Several studies have reported decrease in diabetes incidence following one injection of DCs whose cytokine profile was modulated to produce IL-10 and not IL-12 [48–50]. Since the lifespan of DCs, once injected *in vivo*, is around 12–15 days, it is safe to assume that these DCs may be able to promote long-term tolerance or immunity according to their cytokine production profiles. Indeed, IL-10-producing mature DCs expressing high levels of B7-1 and B7-2 have been shown to induce the development of Tr1 cells that can inhibit inflammation in a model of asthma [51]. A recent study has also shown that injection of GM-CSF into prediabetic NOD mice prevents diabetes development by inducing IL-10-producing tolerogenic DCs that sustain the suppressive function of CD4^+^CD25^+^ regulatory T cells [52]. Similarly, DCs harvested from the pancreatic LN of NOD mice do not produce IL-12 and are tolerogenic as reflected by the protection mediated by their transfer [48]. There is also a possibility that a regulatory Th2 response has been induced after transfer of *L. casei*-treated DCs as found previously after transfer of BM-DCs generated in the presence of GM-CSF/IL-4 [53] or splenic Flt-3L-derived DCs [50]. Conversely, we found that LTA-treated DCs produced much more IL-12 than IL-10, and without upregulating high expression of B7-1 or B7-2 accelerated diabetes when injected into NOD mice. Therefore, our data appear to show that the profile of cytokines produced by DCs may play a determinant role in their ability to induce a pathogenic versus a tolerogenic response. 

The role that IL-10 plays in diabetes development in NOD mice is complex, since IL-10 appears to be involved in diabetes pathogenesis as well as diabetes prevention. On the one hand, there is evidence that IL-10 is involved at some level in diabetes pathogenesis. First, neutralization of IL-10 with anti-IL-10 antibodies effectively blocks insulitis development in NOD mice [54]. In addition, the incidence of diabetes is enhanced in transgenic IL-10-NOD mice expressing IL-10 in the glucagon-producing *α*-cells of the pancreas [55], and adoptive transfers of prediabetic or diabetic wild-type NOD splenocytes into these transgenic IL-10-NOD mice accelerates diabetes [56]. Interestingly, injection of IL-10-deficient NOD splenocytes into transgenic IL-10-NOD. SCID mice accelerates diabetes, demonstrating that pancreatic IL-10, not peripheral IL-10, appears to play a role in diabetes pathogenesis [56]. On the other hand, IL-10 appears to also play an important role in diabetes prevention. Intraperitoneal injection of a long-lived noncytolytic murine IL-10/Fc fusion protein into young NOD mice prevents insulitis and diabetes by blocking the production of proinflammatory cytokines and IFN*γ* [57], and IL-10-transduced islet-specific CD4^+^ T-cells prevent diabetes transfer in NOD mice [58]. Furthermore, systemic delivery of IL-10 by intramuscular injection of expression plasmid DNA can prevent diabetes in NOD mice [59]. Finally, prevention of diabetes by injection of IL-10 expressing vector into NOD mice is associated with an increase in CD4^+^CD25^+^ regulatory T cells [60]. Taken together, these data, and our data suggest that timing, location, and concentration of IL-10 may determine whether IL-10 is protective or disease exacerbating. Our data suggest that IL-10 produced by *Lactobacilli*-treated NOD BM-DCs is involved in the protection observed in our model system and could be having a direct impact or acts via induction of regulatory T cells as shown previously [60, 61]. We attempted to block the protective effect induced by *Lactobacilli*-treated NOD DCs injected into NOD mice using anti-IL10R antibodies *in vivo*. There was a decrease in protection in mice treated with the anti-IL-10R antibodies when compared to mice treated with isotype control, confirming a role for IL-10 in diabetes protection mediated by injection of *Lactobacilli*-treated DCs. Although IL10-deficient NOD mice are not readily available, it would be interesting to test whether BM-DCs from IL-10-deficient NOD mice are capable of preventing diabetes, or whether feeding *Lactobacilli* to IL-10-deficient NOD mice can still affect diabetes development.

*Lactobacilli* have been shown to mediate their effects via TLR-2 [30, 62], possibly via Lipoteichoic acid (LTA), a TLR-2 agonist and one of the main immunostimulatory components of *Lactobacilli* bacteria. However, in contrast to the whole *Lactobacillus *organism, we have found that LTA isolated from *S. aureus* induced NOD BM-DCs to produce much more IL-12 than IL-10 and upregulated the expression of B7-1 and B7-2 on the surface of NOD BM-DCs. Consequently, NOD BM-DCs treated with LTA from *S. aureus* failed to protect NOD mice from diabetes and, in fact, appeared to exacerbate disease. This is not surprising since IL-12 have been reported to mediate disease development in NOD mice via activation of T cells producing IFN*γ* [2, 12, 13]. Analysis of mutant *L. plantarum* that exhibit altered teichoic acid biosynthesis indicates that alteration in LTA structure correlates with a change in its ability to induce proinflammatory versus anti-inflammatory responses [63]. Although we cannot rule out that components other than LTA may be involved in the induction of IL-10 by *Lactobacilli*, the predominant production of IL-12 versus IL-10 by NOD BM-DCs stimulated with LTA isolated from *S. aureus* could be due to differences in the structure of LTA by comparison to *Lactobacilli*, in general, and could also contribute to the differences in cytokine production between the different strains of *Lactobacilli* [10, 11]. It would be interesting to determine whether LTA isolated from *Lactobacilli* behave differently than LTA isolated from *S. aureus*.

## 5. Conclusions

Our data indicate that transfer of differentially stimulated DCs into NOD mice can accelerate or prevent diabetes development depending on the cytokine profile of the DCs. Depending on the strain of *Lactobacilli* and the dose, DCs can be induced to secrete anti-inflammatory cytokines that appear to mediate some diabetes protection. The specific molecular component from *Lactobacilli* that affords protection in NOD mice remains unknown. It will, therefore, be very important to determine the nature of the putative protective component(s) that preferentially induces tolerance versus inflammation, as well as the signalling pathways involved in this process. With this knowledge in hand, it should be possible to design more efficacious DCs-based therapies capable of completely preventing or treating disease. Furthermore, by using pancreatic antigens along with this strategy, it may be possible to generate tolerogenic DCs that are capable of inducing potent antigen-specific regulatory cells and, therefore, are more efficacious in preventing or treating disease.
